# The effect of regional white matter hyperintensities on essential tremor subtypes and severity

**DOI:** 10.3389/fnagi.2022.933093

**Published:** 2022-10-17

**Authors:** Runcheng He, Yan Qin, Xun Zhou, Zhenhua Liu, Qian Xu, Jifeng Guo, Xinxiang Yan, Beisha Tang, Sheng Zeng, Qiying Sun

**Affiliations:** ^1^Department of Geriatric Neurology, Xiangya Hospital, Central South University, Changsha, China; ^2^Department of Neurology, Xiangya Hospital, Central South University, Changsha, China; ^3^Department of Radiology, Xiangya Hospital, Central South University, Changsha, China; ^4^National Clinical Research Center for Geriatric Disorders, Xiangya Hospital, Central South University, Changsha, China; ^5^Key Laboratory of Hunan Province in Neurodegenerative Disorders, Central South University, Changsha, China; ^6^Department of Geriatrics, The Second Xiangya Hospital, Central South University, Changsha, China

**Keywords:** essential tremor, white matter hyperintensities, subtypes, severity, non-motor symptom

## Abstract

**Objectives:**

To investigate the effect of regional white matter hyperintensities (WMHs) on Essential tremor (ET) subtypes and to explore the association between WMHs load and the severity of motor and non-motor symptoms in patients with ET.

**Methods:**

A cohort of 176 patients with ET (including 86 patients with pure ET and 90 patients with ET plus) and 91 normal controls (NC) was consecutively recruited. Demographic, clinical, and imaging characteristics were compared between individuals with pure ET, ET plus, and NC. The cross-sectional association among regional WMHs and the severity of tremor and non-motor symptoms were assessed within each group.

**Results:**

Compared with the pure ET subgroup, the ET plus subgroup demonstrated higher TETRAS scores, NMSS scores, and lower MMSE scores (all *P* < 0.05). Periventricular and lobar WMHs' loads of pure ET subgroup intermediated between NC subjects and ET plus subgroup. WMHs in the frontal horn independently increased the odds of ET (OR = 1.784, *P* < 0.001). The age (*P* = 0.021), WMHs in the frontal lobe (*P* = 0.014), and WMHs in the occipital lobe (*P* = 0.020) showed a significant impact on TETRAS part II scores in the ET plus subgroup. However, only the disease duration was positively associated with TETRAS part II scores in patients with pure ET (*P* = 0.028). In terms of non-motor symptoms, NMSS scores of total patients with ET were associated with disease duration (*P* = 0.029), TETRAS part I scores (*P* = 0.017), and WMH scores in the frontal lobe (*P* = 0.033). MMSE scores were associated with age (*P* = 0.027), body mass index (*P* = 0.006), education level (*P* < 0.001), and WMHs in the body of the lateral ventricle (*P* = 0.005).

**Conclusion:**

Our results indicated that the WMHs in the frontal horn could lead to an increased risk of developing ET. WMHs may be used to differentiate pure ET and ET plus. Furthermore, WMHs in the frontal and occipital lobes are strong predictors of worse tremor severity in the ET plus subgroup. Regional WMHs are associated with cognitive impairment in patients with ET.

## Introduction

Essential tremor (ET) is a common movement disorder affecting ~0.9% of individuals worldwide (Louis and McCreary, 2021). During the last two decades, the conceptualization of ET has evolved over time. The International Parkinson and Movement Disorder Society (IPMDS) formulated a new diagnostic standard for ET and proposed the concept of “ET plus” in 2018 (Bhatia et al., 2018). ET was adopted to describe tremor syndrome with isolated action tremors of bilateral upper limbs of at least 3 years duration. “ET plus” was defined as ET in the presence of additional “soft neurological” signs of uncertain significance, such as memory impairment, questionable dystonic posturing, and impaired tandem gait (Bhatia et al., 2018). Although the etiology of ET remains unclear, an increasing number of evidence has shown that pathological oscillations within the cerebello-thalamo-cortical circuit play an important role in its pathogenesis.

White matter hyperintensities (WMHs), also known as white matter lesions (WMLs), are characterized mainly by hyperintensities on T2-weighted imaging (T_2_WI) and fluid-attenuated inversion recovery (FLAIR) images (Gouw et al., 2011). WMHs are mainly a consequence of axonal loss and demyelination due to chronic ischemia and blood-brain barrier dysfunction (Lin et al., 2017). According to magnetic resonance imaging (MRI) features, WMHs can be classified into lobar WMHs and periventricular WMHs (Noh et al., 2014). In the healthy population, the prevalence of WMHs is ~20% at 60 years, and their prevalence increases exponentially with age (Smith et al., 2017). WMHs are closely linked to cognitive impairment, dementia, urinary incontinence, gait disturbance, and motor compromise in elderly individuals (Gouw et al., 2011; Levit et al., 2020).

WMHs have been associated with several neurodegenerative diseases, including Parkinson's disease (PD) and Alzheimer's disease (AD) (Butt et al., 2021; Gaubert et al., 2021). Two studies to date have found that WMH is associated with ET (Oliveira et al., 2012; Becktepe et al., 2021). However, it is still unclear whether WMHs have different impacts on ET subtypes, such as pure ET and ET plus. Furthermore, the relationship between regional WMHs and the severity of motor and non-motor symptoms in patients with ET has not yet been clarified. The aim of our study was to investigate whether regional WMHs are associated with ET subtypes and to examine the relationship between WMHs load and severity of motor and non-motor symptoms among patients with ET.

## Methods

### Subjects

Our study was conducted on 176 patients with ET and 91 normal controls (NC) consecutively recruited from the inpatients and outpatients of the Department of Neurology, Xiangya Hospital of Central South University, between 1 September 2020 and 30 December 2021 on Parkinson's Disease & Movement Disorders Multicenter Database and Collaborative Network in China (PD-MDCNC, http://pd-mdcnc.com). Ethical approval was obtained from the Medical Ethics Committee of Xiangya Hospital. NC was neurologically normal on the exam. They have never had a history of neurologic, psychiatric, or other major medical illnesses. Diagnosis of ET was confirmed by at least two experienced neurologists according to the 2018 International Movement Disorder Society Tremor Group's essential tremor diagnostic criteria. Patients with ET with any of the following clinical characteristics: questionable dystonic postures, impaired tandem gait, memory impairment, rest tremor, or mild neurological signs of unknown significance were diagnosed with ET plus. Other patients with ET without these symptoms were classified as pure ET. Patients were excluded from our study if they had (1) tremor associated with other central nervous system diseases, such as PD, dystonic tremor, Wilson disease (WD), stroke, and encephalitis; (2) tremor associated with physiological tremor, orthostatic tremor, isolated vocal tremor, and task-specific tremor; and (3) other secondary tremor syndromes.

### Assessments

Demographic and clinical data including gender, age, body mass index (BMI), family history, age at onset (AAO), disease duration, motor symptoms, and non-motor symptoms were collected and entered into the database. Vascular risk factors, such as smoking, hypertension, diabetes, and hyperlipidemia, were also assessed. All patients underwent neurological examinations and neuropsychological assessments.

Tremor severity was evaluated by the Tremor Research Group Essential Tremor Rating Assessment Scale (TETRAS) (Elble et al., 2012). TETRAS part I (items 1–12) was used to evaluate tremor's impact on activities of daily living (0–4 scoring). TETRAS part II (items 1–9) was used to evaluate tremor types (including postural and kinetic tremor), distributions (including head, face, voice, limbs, and trunk tremors), and tremor severity. The Non-Motor Symptoms Scale (NMSS) was used to evaluate the severity of non-motor symptoms. Cognitive function was evaluated by the Mini-Mental State Examination (MMSE).

### MRI scans and WMH grading

**Figure 1:**
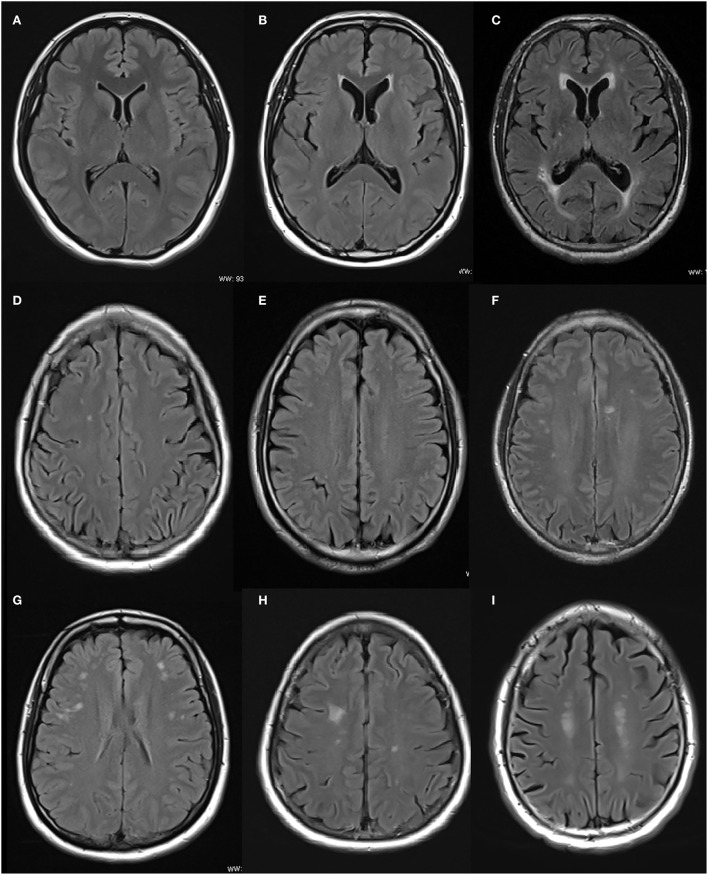
Examples of axial FLAIR images of WMHs' score in frontal horn and frontal lobe. **(A)** Scored 0 point since there were no PWMHs in the frontal horn and occipital horn. **(B)** Scored 1 point since the PWMHs in the frontal horn and occipital horn were <5 mm. **(C)** Scored 2 points since the PWMHs in the frontal horn and occipital horn were between 5 and 10 mm. **(D)** Scored 1 point since the number of lesions in the frontal lobar was <5 and the largest one was smaller than 3 mm. **(E)** Scored 2 points since the number of lesions in the frontal lobar was more than 5 and the largest one was smaller than 3 mm. **(F)** Scored 3 points since the number of lesions in the frontal lobar was <5 and measured between 4 and 10 mm. **(G)** Scored 4 points since the number of lesions in the frontal lobar was more than 6 and measured between 4 and 10 mm. **(H)** Scored 5 points since there was a lesion measured larger than 11 mm. **(I)** Scored 6 points since there were confused lesions in the frontal lobar.

All recruits including patients with ET and NC underwent an MRI examination. Additionally, all MRI images were acquired using a 3.0-T scanner (Prisma, Siemens Healthcare, Erlangen, Germany), and a 64-channel head receiver coil was used. The FLAIR sequence images (repetition time/echo time/inversion time, 9,000/83/2,500 ms; section thickness 5 mm) were used for scoring. Periventricular WMHs and lobar WMHs were evaluated separately according to the semiquantitative visual rating system proposed by Scheltens (Scheltens et al., 1993). Periventricular WMHs in the frontal horn, occipital horn, and the body of the lateral ventricle were scored as 0 point (absent), 1 point ( ≤5 mm), or 2 points (5–10 mm), and total periventricular WMHs score (0–6 points) was calculated. Lobar WMHs in the frontal, parietal, temporal, and occipital lobes were scored as 0 point (absent), 1 point ( ≤5 lesions measuring <3 mm), 2 points (≥6 lesions measuring <3 mm), 3 points ( ≤5 lesions measuring 4–10 mm), 4 points (≥6 lesions measuring 4–10 mm), 5 points (≥1 lesion measuring ≥11 mm), or 6 points (confused lesions), and the total lobar WMHs score (0–24 points) was calculated (Refer to Figure 1). WMHs were scored blindly by two radiologists with more than 3 years of neuroimaging diagnostic experience, and interrater reliability was evaluated by calculating the intraclass correlation coefficient (ICC). If the score was discordant between raters, the final score was determined by consensus. Participants who had lesions such as cerebral hemorrhage, infarction, infection, or brain tumor on MRI were excluded from the study. Images that failed quality control because of movement artifacts of patients with ET also were excluded.

### Statistical analysis

Continuous variables were expressed as mean ± standard error, while percentages and frequencies were used for describing categorical variables. Values were expressed as median (Q1, Q3) for the Scheltens scale. Differences in demographic characteristics among pure ET subgroup, ET plus subgroup, and NC subjects were compared by the analysis of variance with the Kruskal–Wallis test (for non-parametric variables) or the Tukey's test as a *post-hoc* analysis (for parametric variables). Clinical characteristics were compared using the general linear mode. Age, gender, disease duration, smoking status, presence of hypertension, presence of hyperlipidemia, and presence of diabetes were included as confounding variables. The Mann–Whitney *U* test was used to compare the WMHs rated by the Scheltens scale. Logistic regression analysis was used to evaluate the factors associated with ET. Partial correlation analysis was performed to study the relationship between WMHs and tremor severity (TETRAS part I and TETRAS part II scores). Multiple linear regression models were compiled to examine whether WMHs are associated with tremor severity and non-motor symptoms in patients with ET. *P* < 0.05 were considered statistically significant. All analyses were performed using SPSS for Windows Version 24.0 (IBM).

## Results

### Clinical characteristics

The characteristics of the participants are shown in Table 1. Using the new consensus statement of tremor classification, 90 of the 176 ET patients with additional “soft neurological signs” of uncertain significance (e.g., questionable dystonic postures, impaired tandem gait, etc.) were labeled as ET plus subgroup, others (*n* = 86) were classified as pure ET subgroup. Patients with ET plus were older than pure ET and NC subjects (*P* = 0.019, *P* < 0.001, respectively). The AAO of patients with ET plus was significantly older than pure ET (*P* = 0.032). There were no significant differences among the three groups in terms of gender, BMI, family history of tremor, smoking status, hypertension, diabetes, and hyperlipidemia (all *P* > 0.05). Patients with ET plus had higher TETRAS part II scores (*P* = 0.020) and NMSS scores (*P* = 0.002), but lower MMSE scores (*P* < 0.001) than patients with pure ET even after correction for gender, age, disease duration, smoking status, and presence of hypertension, hyperlipidemia, and diabetes. TETRAS part I scores were not statistically different between pure ET and ET plus subgroups (*P* = 0.066).

**Table 1:** Characteristics of the study population (*N* = 267).

	**ET plus subtype**	**Pure ET subtype**	**NC**
	**(*n* = 90)**	**(*n* = 86)**	**(*n* = 91)**
**Demographic characteristics**			
Male, *n* (%)	40 (44.4%)	47 (54.7%)	44 (47.8%)
Age (year)	58.38 ± 15.71^*^^↑^	53.58 ± 14.90	53.62 ± 8.93
BMI (kg/m^2^)	22.91 ± 3.94	23.52 ± 3.31	23.12 ± 3.63
Education (y)	9.38 ± 4.56^*^	10.98 ± 4.44	–
AAO (y)	48.27 ± 17.78^*^	43.71 ± 16.23	–
Duration (y)	10.11 ± 10.84	9.87 ± 9.29	–
Family history of tremor (%)	47 (52.2%)	50 (58.1%)	–
Smoking	14 (15.6%)	18 (20.9%)	18 (19.8%)
Hypertension	16 (17.8%)	25 (29.1%)	21 (23.1%)
Diabetes	8 (8.9%)	6 (7.0%)	9 (9.9%)
Hyperlipidemia	16 (17.8%)	19 (22.1%)	17 (18.7%)
**Clinical characteristics**			
TETRAS part I	16.12 ± 9.64^*^	12.40 ± 9.96	–
TETRAS part II	20.47 ± 8.30^*^	16.99 ± 7.53	–
NMSS	17.00 ± 19.73^*^	8.50 ± 12.34	–
MMSE	26.64 ± 3.16^*^	28.45 ± 1.72	–
**Neuroimaging characteristics**			
Periventricular WMHs scores	3 (2,3.75)^*^^↑^	2 (0,3)^↑^	0 (0,2)
Frontal horn	1 (1,1)^*^^↑^	1 (0,1)^↑^	0 (0,1)
Occipital horn	1 (1,1)^*^^↑^	1 (0,1)^↑^	0 (0,1)
The body of the lateral ventricle	1 (0,1)^*^^↑^	0 (0,1)^↑^	0 (0,0)
Lobar WMHs score	4 (1,6.75)^*^^↑^	1 (0,2)^↑^	0 (0,1)
Frontal lobe	2 (1,3)^*^^↑^	1 (0,1)^↑^	0 (0,1)
Parietal lobe	1 (0,3)^*^^↑^	0 (0,1)	0 (0,0)
Temporal lobe	0 (0,1)^↑^	0 (0,0)	0 (0,0)
Occipital lobe	0 (0,0)^↑^	0 (0,0)	0 (0,0)

* P < 0.05, vs. Pure ET.

↑ P < 0.05, vs. NC.

ET, essential tremor; NC, normal controls; BMI, body mass index; AAO, age at onset; TETRAS, Tremor Research Group Essential Tremor Rating Assessment Scale; NMSS, non-motor symptom assessment scale; MMSE, mini-mental state examination; WHM, white matter hyperintensity.

### Effects of regional WMHs on ET subtypes

**Figure 2:**
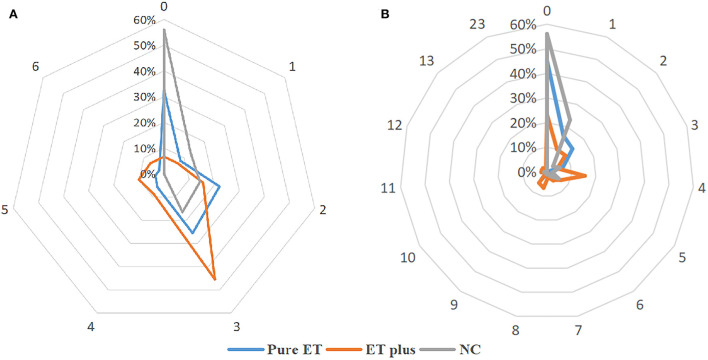
The distribution frequency of periventricular WMHs' scores **(A)** and lobar WMHs' scores **(B)**.

**Figure 3:**
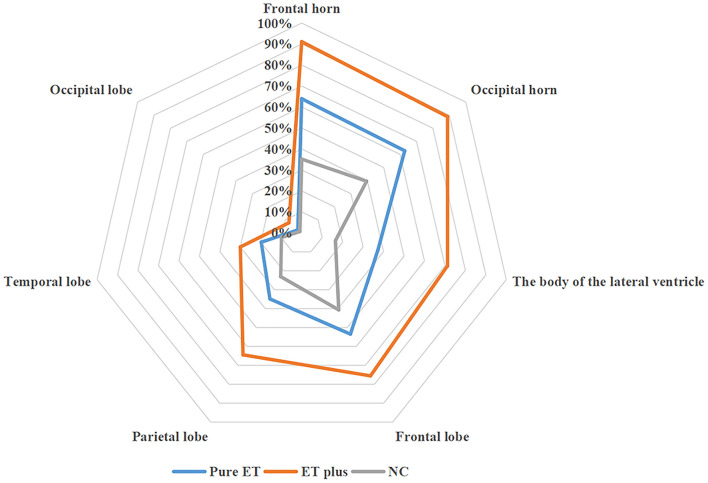
The distribution frequency of WMHs in different brain regions among participants.

Compared to the NC group, pure ET subtype had higher WMHs' load in the frontal horn, occipital horn, the body of the lateral ventricle, frontal lobe, and parietal lobe (all *P* < 0.05). The WMHs' loads in the frontal horn, occipital horn, the body of the lateral ventricle, frontal lobe, and parietal lobe of the ET plus subtype were higher than those of the pure ET subtype (all *P* < 0.05) (Table 1). Regarding MR imaging characteristics, the distribution frequency of periventricular WMHs' scores (*P* < 0.001) and lobar WMHs' scores (*P* < 0.001) are shown in Figure 2. Periventricular WMHs' scores and lobar WMHs' scores in the ET plus subgroup varied across a wider range than those in the pure ET subgroup and NC group. As shown in Figure 3, WMHs in all brain lobes appeared most frequently in the ET plus subtype, followed by the pure ET subtype and NC group (*P* < 0.001). Binary logistic regression analysis was used to investigate whether the impact of WMHs on the development of ET would depend on the regional WMH distribution (Table 2). The results indicated that WMHs in the frontal horn (OR = 1.784, *P* < 0.001) were significantly associated with ET occurrence.

**Table 2:** Binary logistic regression analysis of factors associated with NC and ET.

**Variables**	**Univariate**	**Multivariate**	**Multivariate**
	***P*** -value*	**OR (95% CI)**	***P*** **-value**
Gender	0.736	–	Not included
Age	0.210	–	Not included
Duration (y)	–	–	Not included
BMI	0.851	–	Not included
Smoking	0.595	–	Not included
Hypertension	0.968	–	Not included
Diabetes	0.594	–	Not included
Hyperlipidemia	0.437	–	Not included
Frontal horn	**<0.001**	1.784	**<0.001**
Occipital horn	**<0.001**	–	0.561
The body of the lateral ventricle	**<0.001**	–	0.086
Periventricular WMHs scores	**<0.001**	–	0.143
Frontal lobe	**<0.001**	–	0.321
Parietal lobe	**<0.001**	–	0.603
Temporal lobe	**0.009**	–	0.917
Occipital lobe	0.172	–	Not included
Lobar WMHs score	**0.029**		0.463

Significant P-values are in bold.

OR, odd ratio; NC, normal controls; ET, essential tremor; BMI, body mass index; WHM, white matter hyperintensity.

### Relationship between regional WMHs and tremor severity

We examined the relationship between WMHs' load and tremor severity in total ET, pure ET subtype, and ET plus subtype, after adjusting for gender, age, BMI, and disease duration (Table 3). The partial correlation analysis showed that TETRAS part I scores were significantly associated with frontal horn scores (*r*_*s*_ = 0.219, *P* = 0.004), occipital horn scores (*r*_*s*_ = 0.160, *P* = 0.035), frontal lobe scores (*r*_*s*_ = 0.194, *P* = 0.011), and TETRAS part II scores were associated with frontal horn scores (*r*_*s*_ = 0.155, *P* = 0.042), frontal lobe scores (*r*_*s*_ = 0.243, *P* = 0.001), parietal lobe scores (*r*_*s*_ = 0.180, *P* = 0.018), and occipital lobe (*r*_*s*_ = 0.245, *P* = 0.001) in total patients with ET. In the pure ET subtype, TETRAS part I scores were significantly associated with occipital horn scores (*r*_*s*_ = 0.231, *P* = 0.037) and occipital lobe scores (*r*_*s*_ = 0.249, *P* = 0.024). TETRAS part II scores were associated with frontal lobe scores (*r*_*s*_ = 0.218, *P* = 0.049), parietal lobe scores (*r*_*s*_ = 0.261, *P* = 0.018), and occipital lobe scores (*r*_*s*_ = 0.235, *P* = 0.033). In ET plus, neither periventricular WMHs nor lobar WMHs' scores were associated with TETRAS part I scores. TETRAS part II scores were associated with frontal lobe scores (*r*_*s*_ = 0.213, *P* = 0.049) and occipital lobe scores (*r*_*s*_ = 0.267, *P* = 0.013) in the ET plus subtype.

**Table 3:** Association of WMHs and tremor severity in total ET, pure ET subtype, and ET plus subtype.

	**Frontal**		**Occipital**		**The body of the**		**Frontal**		**Parietal**		**Temporal**		**Occipital**	
	**horn**		**horn**		**lateral ventricle**		**lobe**		**lobe**		**lobe**		**lobe**	
	***r_*s*_***	***P***	***r_*s*_***	***P***	***r_*s*_***	***P***	***r_*s*_***	***P***	***r_*s*_***	***P***	***r_*s*_***	***P***	***r_*s*_***	***P***
**Total ET**														
TETRAS part I	0.219	**0.004**	0.160	**0.035**	0.141	0.065	0.194	**0.011**	0.104	0.175	0.051	0.504	0.104	0.174
TETRAS part II	0.155	**0.042**	0.105	0.169	0.144	0.059	0.243	**0.001**	0.180	**0.018**	0.114	0.136	0.245	**0.001**
**Pure ET**														
TETRAS part I	0.213	0.055	0.231	**0.037**	0.156	0.162	0.167	0.134	0.138	0.215	0.158	0.155	0.249	**0.024**
TETRAS part II	0.160	0.150	0.179	0.107	0.104	0.352	0.218	**0.049**	0.261	**0.018**	0.121	0.280	0.235	**0.033**
**ET plus**														
TETRAS part I	0.128	0.242	−0.018	0.868	0.057	0.601	0.156	0.152	0.041	0.707	−0.052	0.635	0.061	0.574
TETRAS part II	0.074	0.498	−0.061	0.576	−0.091	0.406	0.213	**0.049**	0.88	0.418	0.095	0.383	0.267	**0.013**

Significant P-values are in bold.

ET, essential tremor; BMI, body mass index; TETRAS, Tremor Research Group Essential Tremor Rating Assessment Scale.

The results of the multivariate regression models used for evaluating the associations between tremor severity (TETRAS part II) and WMHs are shown in Table 4. Statistical analysis revealed that older age (*β* = 0.186, *P* = 0.036) and higher occipital lobe scores (*β* = 0.186, *P* = 0.015) were significantly associated with higher TETRAS part II scores in total patients with ET. In patients with pure ET, only the disease duration (*β* = 0.237, *P* = 0.028) was a significant factor in increasing the tremor severity (TETRAS part II), while age and WMHs' scores were not significant (all *P* > 0.05). In patients with ET plus, age (*β* = 0.289, *P* = 0.021), frontal lobe scores (*β* = 0.521, *P* = 0.014), and occipital lobe scores (*β* = 0.256, *P* = 0.020) showed significant impacts on tremor severity (TETRAS part II), while disease duration was not significant (all *P* > 0.05).

**Table 4:** Multivariable linear regression analysis of clinical and imaging factors associated with tremor severity (TETRAS part II) in total ET, pure ET subtype, and ET plus subtype.

	**Total**		**Pure ET**		**ET plus**	
	**ET**		**subtype**		**subtype**	
	**Beta**	***P***	**Beta**	***P***	**Beta**	***P***
Gender	0.053	0.457	0.108	0.294	0.085	0.418
Age	0.186	**0.036**	0.134	0.305	0.289	**0.021**
BMI	−0.096	0.179	−0.182	0.098	−0.020	0.843
Duration (y)	0.100	0.169	0.237	**0.028**	0.226	0.822
Frontal horn scores	0.110	0.336	−0.085	0.667	0.156	0.246
Occipital horn scores	−0.060	0.593	0.189	0.287	−0.282	0.051
Frontal lobe scores	0.283	0.062	−0.089	0.683	0.521	**0.014**
Parietal lobe scores	−0.143	0.337	0.285	0.172	−0.410	0.057
Occipital lobe scores	0.186	**0.015**	0.202	0.064	0.256	**0.020**

Significant P-values are in bold.

ET, essential tremor; BMI, body mass index; TETRAS, Tremor Research Group Essential Tremor Rating Assessment Scale.

### Factors correlated with non-motor symptoms in ET

Regression models using NMSS score as an independent variable revealed that higher NMSS scores in total patients with ET were associated with longer disease duration (*β* = 0.161, *P* = 0.0293), higher TETRAS part I scores (*β* = 0.149, *P* = 0.017), and higher WMHs' scores in the frontal lobe (*β* = 0.161 *P* = 0.033). In addition, MMSE scores were significantly associated with age (*β* = −0.173, *P* = 0.027), BMI (*β* = 0.170, *P* = 0.006), education level (*β* = 0.300, *P* < 0.001), and WMHs in the body of the lateral ventricle (*β* = −0.276, *P* = 0.005) (Table 5).

**Table 5:** Multivariable linear regression analysis of NMSS and MMSE scores associated with clinical and imaging factors in total patients with ET.

	**NMSS**		**MMSE**	
	**Beta**	***P***	**Beta**	***P***
Gender	−0.094	0.193	0.074	0.237
Age	−0.071	0.386	−0.173	**0.027**
BMI	−0.085	0.240	0.170	**0.006**
Education (y)	−0.089	0.258	0.300	**<0.001**
Duration (y)	0.161	**0.029**	−0.034	0.589
TETRAS part I	0.184	**0.017**	−0.182	0.102
TETRAS part II	−0.027	0.832	0.572	0.599
Frontal horn	0.038	0.679	−0.022	0.723
Occipital horn	0.087	0.320	−0.199	0.050
The body of the lateral ventricle	0.031	0.742	−0.276	**0.005**
Frontal lobe	0.161	**0.033**	0.247	0.074
Parietal lobe	−0.066	0.662	−0.228	0.089
Temporal lobe	−0.088	0.339	0.014	0.903
Occipital lobe	0.052	0.494	0.019	0.837

Significant P-values are in bold.

NMSS, Non-motor symptom assessment scale; MMSE, Mini-Mental State Examination; BMI, body mass index; TETRAS, Tremor Research Group Essential Tremor Rating Assessment Scale.

## Discussion

To the best of our knowledge, this is the first study to explore the effects of regional WMHs on the ET subtypes, tremor severity, and non-motor symptoms in pure ET subtype and ET plus subtype.

Our results were in agreement with previous neuroimaging reports that both periventricular WMHs and lobar WMHs are larger in patients with ET compared with NC subjects (Oliveira et al., 2012; Becktepe et al., 2021). WMHs may disturb white matter fibers that connect the cortex to subcortical nuclei and other distant brain territories, resulting in structural and functional abnormalities of neural circuits and eventually leading to tremor (Zhu et al., 2021). Several electrophysiological and imaging studies have strongly confirmed the major role of the cerebello-thalamo-cortical network in the pathogenesis of ET (Schnitzler et al., 2009; Lenka et al., 2017; Nicoletti et al., 2020). Furthermore, a large number of studies confirmed the important role of a neural circuit involving frontal and parietal areas in movement sequencing (Bortoletto and Cunnington, 2010; Benito-León et al., 2018). The frontoparietal network was thought to be associated with ET (Oliveira et al., 2012; Benito-León et al., 2015; Cao et al., 2018). Fibers from the corpus callosum and corona radiata run through the frontal horn, and WMHs in the frontal horn might relate to the disruption of neural loops and potentially affect frontal lobe function (Toda et al., 2019; Louis et al., 2020). Our study provides important evidence supporting the diagnostic significance of WMHs in ET. We found that the presence of WMHs in the frontal horn was a significant predictor of ET. As we all know, severe WMHs are associated with cognitive impairment, gait impairment, and neuropsychiatric disorders. Compared to the pure ET subtype, our research indicates that ET plus subtype with soft neurological signs has higher WMHs' load. WMHs' load may be used to differentiate pure ET and ET plus.

Our study suggested that there might be some heterogenicity in different subtypes of ET. Yet no other studies have explored the association between WMHs and the subtypes of ET. Our study for the first time suggested that patients with ET plus subtype have higher TETRAS scores and carried more serious WMHs in five regions than pure ET subtype after adjusting for age, disease duration, and vascular risk factors. Our study found a predominant association among WMHs in frontal, and tremor severity in patients with ET plus subtype compared with pure ET. The cortical changes in the caudal middle frontal gyrus are thought to be associated with tremor, as this region act on motor output through direct or indirect connections with the primary motor cortex and the spinal cord (Picard and Strick, 2001; Karabanov et al., 2012). WMHs in the occipital lobe may also be involved in some way with the modulation of tremor. Visual association areas have been implicated to be involved in tremor generation (Tuleasca et al., 2018). Lobar WMHs in the occipital lobe may interrupt the occipital cortical interaction with the frontal cortex (Kravitz et al., 2011; Wan et al., 2019). Moreover, older age was linked with a more severe tremor in patients with ET plus. In our opinion, tremor severity is exacerbated by increased WMHs in the frontal and occipital lobes in patients with ET plus. Patients with pure ET whose clinical symptoms and WMHs' loads were milder than ET plus were found only to have an association between disease duration and tremor severity, while WMHs had no significant effect. Tremor severity is exacerbated by increased disease duration in patients with pure ET.

We found that patients with the ET plus subtype presented a more serious cognitive impairment and more severe non-motor symptoms, compared to the patients of the pure ET subtype. Non-motor symptoms in patients with ET often worsen as the disease duration increases, exacerbated tremor severity, and increased WMHs in the frontal lobe. Frontal lobe function has been considered to be directly associated with non-motor symptoms (Kwon et al., 2016). Impairments in the frontal-cerebellar circuit were identified to be the most prominent risk factor for cognitive decline in patients with ET (Gasparini et al., 2001; Benito-León et al., 2015). Our study pointed toward a direct link between WMHs and cognitive impairment. Cognitive performance negatively correlated with WMHs in the body of the lateral ventricle, but not with lobar WMHs. The damage to neural pathways in the periventricular white matter has more influence on cognitive decline than the damage to subcortical pathways (De Groot et al., 2002; Yoshita et al., 2006). The WMHs in the body of the lateral ventricle disrupt the connection integrity of the temporal lobe with other lobes such as the occipital lobe, which is associated with memory function (Chen et al., 2020). The WMHs in the body of the lateral ventricle are a prelude to cognitive deficits in patients with ET. As a result, preventing and decreasing the periventricular WMHs can be an important strategy to prevent cognitive impairment in patients with ET.

Our study identified WMHs in the frontal horn as a significant contributor to the neuropathogenesis of ET. The pathology of WMHs might not be involved in the pathophysiology of ET subtypes. We further demonstrated that age, WMHs in the frontal, and occipital lobe are associated with worse tremor severity in the ET plus subgroup. We also found a close association between the non-motor symptoms and disease duration, TETRAS part I scores, and WMHs' load in the frontal lobe. Given that WMH has several modifiable vascular risk factors, interventions aimed at reducing vascular risk factors may be beneficial in reducing tremor symptoms in patients with ET plus. On the other hand, this study has some limitations. Our study was a cross-sectional study, and the sample size was relatively small. Therefore, a larger longitudinal study is needed to reveal the association between WMHs and disease progression in the future.

## Conclusion

We identified that the WMHs in the frontal horn could lead to an increased risk of developing ET. WMHs load may be used to differentiate pure ET and ET plus. Furthermore, our results indicated that age and WMHs in frontal and occipital lobes are strong predictors of worse tremor severity in the ET plus subgroup. WMHs in the body of the lateral ventricle are associated with cognitive impairment in patients with ET.

## Data availability statement

The raw data supporting the conclusions of this article will be made available by the authors, without undue reservation.

## Ethics statement

The studies involving human participants were reviewed and approved by the Medical Ethics Committee of the Xiangya Hospital, Central South University. The participants provided their written informed consent to participate in this study. Written informed consent was obtained from the individuals for the publication of any potentially identifiable images or data included in this article.

## Author contributions

Material preparation and data collection were performed by RH, YQ, SZ, and QS. Data collection analyses were performed by RH, XZ, ZL, QX, XY, JG, BT, SZ, and QS. The first draft of the manuscript was written by RH, YQ, and SZ. All authors contributed to the study's conception, design, commented on previous versions of the manuscript, read, and approved the final manuscript.

## Conflict of interest

The authors declare that the research was conducted in the absence of any commercial or financial relationships that could be construed as a potential conflict of interest.

## Publisher's note

All claims expressed in this article are solely those of the authors and do not necessarily represent those of their affiliated organizations, or those of the publisher, the editors and the reviewers. Any product that may be evaluated in this article, or claim that may be made by its manufacturer, is not guaranteed or endorsed by the publisher.

## References

[B1] Becktepe J. S. Busse J. Jensen-Kondering U. Toedt I. Wolff S. Zeuner K. E.. (2021). White matter hyperintensities are associated with severity of essential tremor in the elderly. Front. Neurol. 12, 694286. 10.3389/fneur.2021.694286 34262526 PMC8273287

[B2] Benito-León J. Louis E. D. Romero J. P. Hernández-Tamames J. A. Manzanedo E. Álvarez-Linera J.. (2015). Altered functional connectivity in essential tremor: a resting-state fMRI study. Medicine 94, e1936. 10.1097/MD.0000000000001936 26656325 PMC5008470

[B3] Benito-León J. Serrano J. I. Louis E. D. Holobar A. Romero J. P. Povalej-BrŽan P.. (2018). Tremor severity in Parkinson's disease and cortical changes of areas controlling movement sequencing: a preliminary study. J. Neurosci. Res. 96, 1341–1352. 10.1002/jnr.24248 29660812

[B4] Bhatia K. P. Bain P. Bajaj N. Elble R. J. Hallett M. Louis E. D.. (2018). Consensus Statement on the classification of tremors from the task force on tremor of the international Parkinson and movement disorder society. Mov. Disord. 33, 75–87. 10.1002/mds.27121 29193359 PMC6530552

[B5] Bortoletto M. Cunnington R. (2010). Motor timing and motor sequencing contribute differently to the preparation for voluntary movement. Neuroimage 49, 3338–3348. 10.1016/j.neuroimage.2009.11.048 19945535

[B6] Butt A. Kamtchum-Tatuene J. Khan K. Shuaib A. Jickling G. C. Miyasaki J. M.. (2021). White matter hyperintensities in patients with Parkinson's disease: a systematic review and meta-analysis. J. Neurol. Sci. 426, 117481. 10.1016/j.jns.2021.117481 33975191

[B7] Cao H. Wang R. Luo X. Li X. Hallett M. Thompson-Westra J.. (2018). A voxel-based magnetic resonance imaging morphometric study of cerebral and cerebellar gray matter in patients under 65 years with essential tremor. Med. Sci. Monit. 24, 3127–3135. 10.12659/MSM.906437 29754151 PMC5973500

[B8] Chen H. F. Huang L. L. Li H. Y. Qian Y. Yang D. Qing Z.. (2020). Microstructural disruption of the right inferior fronto-occipital and inferior longitudinal fasciculus contributes to WMH-related cognitive impairment. CNS Neurosci. Ther. 26, 576–588. 10.1111/cns.13283 31901155 PMC7163793

[B9] De Groot J. C. De Leeuw F. E. Oudkerk M. Van Gijn J. Hofman A. Jolles J.. (2002). Periventricular cerebral white matter lesions predict rate of cognitive decline. Ann. Neurol. 52, 335–341. 10.1002/ana.10294 12205646

[B10] Elble R. Comella C. Fahn S. Hallett M. Jankovic J. Juncos J. L.. (2012). Reliability of a new scale for essential tremor. Mov. Disord. 27, 1567–1569. 10.1002/mds.25162 23032792 PMC4157921

[B11] Gasparini M. Bonifati V. Fabrizio E. Fabbrini G. Brusa L. Lenzi G. L.. (2001). Frontal lobe dysfunction in essential tremor: a preliminary study. J. Neurol. 248, 399–402. 10.1007/s004150170181 11437162

[B12] Gaubert M. Lange C. Garnier-Crussard A. Köbe T. Bougacha S. Gonneaud J.. (2021). Topographic patterns of white matter hyperintensities are associated with multimodal neuroimaging biomarkers of Alzheimer's disease. Alzheimers Res. Ther. 13, 29. 10.1186/s13195-020-00759-3 33461618 PMC7814451

[B13] Gouw A. A. Seewann A. van der Flier W. M. Barkhof F. Rozemuller A. M. Scheltens P.. (2011). Heterogeneity of small vessel disease: a systematic review of MRI and histopathology correlations. J. Neurol. Neurosurg. Psychiatry 82, 126–135. 10.1136/jnnp.2009.204685 20935330

[B14] Karabanov A. Jin S. H. Joutsen A. Poston B. Aizen J. Ellenstein A.. (2012). Timing-dependent modulation of the posterior parietal cortex-primary motor cortex pathway by sensorimotor training. J. Neurophysiol. 107, 3190–3199. 10.1152/jn.01049.2011 22442568 PMC3378371

[B15] Kravitz D. J. Saleem K. S. Baker C. I. Mishkin M. (2011). A new neural framework for visuospatial processing. Nat. Rev. Neurosci. 12, 217–230. 10.1038/nrn3008 21415848 PMC3388718

[B16] Kwon K. Y. Lee H. M. Lee S. M. Kang S. H. Koh S. B. (2016). Comparison of motor and non-motor features between essential tremor and tremor dominant Parkinson's disease. J. Neurol. Sci. 361, 34–38. 10.1016/j.jns.2015.12.016 26810513

[B17] Lenka A. Bhalsing K. S. Panda R. Jhunjhunwala K. Naduthota R. M. Saini J.. (2017). Role of altered cerebello-thalamo-cortical network in the neurobiology of essential tremor. Neuroradiology 59, 157–168. 10.1007/s00234-016-1771-1 28062908

[B18] Levit A. Hachinski V. Whitehead S. N. (2020). Neurovascular unit dysregulation, white matter disease, and executive dysfunction: the shared triad of vascular cognitive impairment and Alzheimer disease. GeroScience 42, 445–465. 10.1007/s11357-020-00164-6 32002785 PMC7205943

[B19] Lin J. Wang D. Lan L. Fan Y. (2017). Multiple factors involved in the pathogenesis of white matter lesions. Biomed. Res. Int. 2017, 9372050. 10.1155/2017/9372050 28316994 PMC5339523

[B20] Louis E. D. Bares M. Benito-Leon J. Fahn S. Frucht S. J. Jankovic J.. (2020). Essential tremor-plus: a controversial new concept. Lancet Neurol. 19, 266–270. 10.1016/S1474-4422(19)30398-9 31767343 PMC10686582

[B21] Louis E. D. McCreary M. (2021). How common is essential tremor? Update on the worldwide prevalence of essential tremor. Tremor Other Hyperkinet. Mov. 11, 28. 10.5334/tohm.632 34277141 PMC8269764

[B22] Nicoletti V. Cecchi P. Pesaresi I. Frosini D. Cosottini M. Ceravolo R. (2020). Cerebello-thalamo-cortical network is intrinsically altered in essential tremor: evidence from a resting state functional MRI study. Sci. Rep. 10, 16661. 10.1038/s41598-020-73714-9 33028912 PMC7541442

[B23] Noh Y. Lee Y. Seo S. W. Jeong J. H. Choi S. H. Back J. H.. (2014). A new classification system for ischemia using a combination of deep and periventricular white matter hyperintensities. J. Stroke Cerebrovasc. Dis. 23, 636–642. 10.1016/j.jstrokecerebrovasdis.2013.06.002 23867045

[B24] Oliveira A. P. Brickman A. M. Provenzano F. A. Muraskin J. Louis E. D. (2012). White matter hyperintensity burden on magnetic resonance imaging in essential tremor. Tremor Other Hyperkinet. Mov. 2, tre-02-28-95-3. 10.5334/tohm.82 23439769 PMC3569979

[B25] Picard N. Strick P. L. (2001). Imaging the premotor areas. Curr. Opin. Neurobiol. 11, 663–672. 10.1016/S0959-4388(01)00266-5 11741015

[B26] Scheltens P. Barkhof F. Leys D. Pruvo J. P. Nauta J. J. Vermersch P.. (1993). A semiquantative rating scale for the assessment of signal hyperintensities on magnetic resonance imaging. J. Neurol. Sci. 114, 7–12. 10.1016/0022-510X(93)90041-V 8433101

[B27] Schnitzler A. Münks C. Butz M. Timmermann L. Gross J. (2009). Synchronized brain network associated with essential tremor as revealed by magnetoencephalography. Mov. Disord. 24, 1629–1635. 10.1002/mds.22633 19514010

[B28] Smith E. E. Saposnik G. Biessels G. J. Doubal F. N. Fornage M. Gorelick P. B.. (2017). Prevention of stroke in patients with silent cerebrovascular disease: a scientific statement for healthcare professionals from the American Heart Association/American Stroke Association. Stroke 48, e44–e71. 10.1161/STR.0000000000000116 27980126

[B29] Toda K. Iijima M. Kitagawa K. (2019). Periventricular white matter lesions influence gait functions in Parkinson's disease. Eur. Neurol. 81, 120–127. 10.1159/000499908 31203285

[B30] Tuleasca C. Najdenovska E. Régis J. Witjas T. Girard N. Champoudry J.. (2018). Pretherapeutic motor thalamus resting-state functional connectivity with visual areas predicts tremor arrest after Thzalamotomy for essential tremor: tracing the cerebello-thalamo-visuo-motor network. World Neurosurg. 117, e438–e449. 10.1016/j.wneu.2018.06.049 29920392

[B31] Wan Y. Hu W. Gan J. Song L. Wu N. Chen Y.. (2019). Exploring the association between Cerebral small-vessel diseases and motor symptoms in Parkinson's disease. Brain Behav. 9, e01219. 10.1002/brb3.1219 30815987 PMC6456802

[B32] Yoshita M. Fletcher E. Harvey D. Ortega M. Martinez O. Mungas D. M.. (2006). Extent and distribution of white matter hyperintensities in normal aging, MCI, and AD. Neurology 67, 2192–2198. 10.1212/01.wnl.0000249119.95747.1f 17190943 PMC3776588

[B33] Zhu W. Huang H. Yang S. Luo X. Zhu W. Xu S.. (2021). Cortical and subcortical grey matter abnormalities in white matter hyperintensities and subsequent cognitive impairment. Neurosci. Bull. 37, 789–803. 10.1007/s12264-021-00657-0 33826095 PMC8192646

